# Castration

**Published:** 1897-02

**Authors:** T. J. Pugh

**Affiliations:** Hearne, Texas


For the Texas Medical Journal.

### CASTRATION.

T. J. PUGH, M. D., HEARNE, TEXAS.

THE subject of castration for the prevention and cure of rape has been quite extensively agitated through your journal.

Dr. De Causey, in an article written for the TEXAS MEDICAL JOURNAL, which appeared in a late issue of that very excellent exponent of medical sentiment, was read by me with more than usual interest.

I agree with the doctor in most of his ideas—and I am glad he has the courage to meet the castration craze which seems to have fastened itself upon the minds of some of our home medical men.

I can but condemn the idea entertained by some members of our profession who advocate castration for the *prevention and cure* of this great crime so-called, which is indeed a disease. How can you *prevent* a thing which has already taken place? These men who suggest castration as a prevention of rape must think the power of evil conception originates in the testicle instead of the brain. Did you ever hear of a blind man committing rape? Then why not job out the eyes of the people generally as a prevention of this revolting deed? The eyes are said to be the "windows of the soul." Truly they are the windows of the brain. The unfortunate, diseased brain sees the object of his evil wish, and in an ungoverned and uncontrollable moment he executes a deed from which he has no moral power to refrain. Would castration *prevent* this deed that has already been committed? Does the rapist deserve death for committing a crime which must be the result of a diseased brain? It is a fearful thing, I admit, to do a thing like this—yet will you punish disease? I hold that a sane man can't commit rape—can't do the deed without the consent of the associated party—any more than a well man can have a chill, fever or pneumonia. Shall we hold a person responsible by death or castration for having a disease over which he has no power of control?

[If rape is not a "crime," what is? You are away off, Doctor, and will find none to agree with you that these burly black brutes who commit rape on white girls are "not responsible," it is brute passion, and is not an "evidence of an unbalanced mind."—ED.]

I favor punishing crime, but disease never. To do such a deed should be evidence of guilt to that extent that the unfortunate should be carefully and at the same time humanely imprisoned, simply as a precaution against a repetition of a like deed. See the revolting deeds done throughout our State and nation, many of which have been ascribed to an undoubtedly diseased or distorted brain.

Insanity causes the insane parent to butcher its helpless and confiding offspring. This is all the result of the same kind of dethroned reason. This kind of murderer often realizes just after the accomplishment of such a deed that wrong has been done, and an effort to conceal the deed is made. The deed has been accomplished while the actor was under a temporary spell of insanity. Possibly reason will again resume its usual balance, and the unfortunate being realizes the enormity of his deed, and fear of punishment causes him to flee, and if caught to deny his guilt.

This is a subject I know that will find but few to defend, yet it is a fact that many regard the brain that would impel one to the commission of such a deed as one unbalanced and diseased. Then if it is a disease, is it right to punish disease with death or castration?

